# Protocol for a randomized controlled trial of steroid versus methotrexate as first-line monotherapy in the management of idiopathic granulomatous mastitis

**DOI:** 10.1371/journal.pone.0333577

**Published:** 2025-10-27

**Authors:** Serene Si Ning Goh, Peter Cheung, Sen Hee Tay, Margaret Ma, Thomas Choudary Putti, Bee Choo Tai, Karen Kaye Casida, Zhi Hua Chua, Ying Jia Chew, Nur Khaliesah Binte Mohamed Riza, Jenny Liu, Mikael Hartman

**Affiliations:** 1 Department of Surgery, National University Hospital and National University Health System, Singapore; 2 Department of Surgery, Yong Loo Lin School of Medicine, National University of Singapore and National University Health System, Singapore; 3 Division of Rheumatology and Allergy, Department of Medicine, National University Hospital, Singapore; 4 Department of Medicine, Yong Loo Lin School of Medicine, National University of Singapore, Singapore; 5 Department of Pathology, Yong Loo Lin School of Medicine, National University of Singapore, Singapore; 6 Saw Swee Hock School of Public Health, National University of Singapore and National University Health System, Singapore; 7 Department of General Surgery, National University Hospital, Singapore; 8 Department of General Surgery, Ng Teng Fong General Hospital, Singapore; University of Colombo Faculty of Medicine, SRI LANKA

## Abstract

**Background:**

Idiopathic granulomatous mastitis (IGM) is a rare inflammatory breast disease affecting mainly young to middle aged women. Its aetiology is idiopathic and poorly understood, but recent evidence supports its autoimmunity pathogenesis. This pathogenesis supports the current first line treatment involving steroid monotherapy or steroid combination therapy with methotrexate, however, there is no single well-established treatment protocol that has been shown to most effectively cure the disease. Thus, this prospective, open, two arm randomised controlled trial comparing the effectiveness of steroid versus methotrexate therapy in the treatment of IGM patients was developed, with the hypothesis that methotrexate has a higher 6-months clinical complete response and radiologically complete response rate than steroid monotherapy.

**Methods:**

This trial will be conducted at the Breast Care Center of the National University Hospital Singapore. To be eligible, patients must have undergone breast ultrasound, biopsy, and histologic diagnosis of IGM. Forty eligible patients between the ages 21–60 will be recruited obtaining informed consent. Baseline blood tests and imaging will be obtained during screening phase. Eligible patients after the screening phase will be randomised to either oral corticosteroid or methotrexate group. Treatment phase commences in which patients in both arms will receive either oral corticosteroid or oral methotrexate over 3 months, with follow-ups conducted at 6- and 12-months post randomisation. Safety measures including reporting of adverse events along the course of the trial will be observed. The primary outcome is 6-month complete clinical or radiological response rate, alongside time to cCR post randomization, treatment failure (TF) rate, and relapse rate (RR). Secondary outcomes include identification of drug adverse effect, frequency of surgical intervention, determination of potential biomarkers, and patient reported outcomes.

**Ethics and Dissemination:**

This study is approved by the ethics committee of the National University Singapore (DSRB reference number 2023/00773). Results of this trial will be shared and presented in local and international scientific conferences, and will be published in international journals.

## Introduction

Idiopathic granulomatous mastitis (IGM) is a rare, benign, chronic inflammatory disease of the breast, often mimics inflammatory breast cancer. It affects 2.4 per 100,000 women with age ranging from 20 to 40 years, with increased incidence in Hispanic and Asian women [1]. Its most common presentation includes breast mass with or without pain, erythema, subcutaneous or deep abscess, sinus tracts or fistula, and axillary lymphadenopathy [2]. The disease has a chronic recurrent course with multiple flares which affects the quality of life in these women who are usually in their reproductive years. It is a disfiguring illness with potential negative psychosocial impact on young women. The diagnosis is established through clinical evaluation; imaging which shares the same features of a carcinoma; and histopathology which involves formation of non-caseating granuloma [3].

As the etiology of IGM is poorly understood, its treatment poses a challenge for breast physicians. Currently, there is no single well-established treatment protocol that has been shown to most effectively cure the disease. Various therapeutic options for IGM have been reported such as antibiotics, steroids, non-steroidal anti-inflammatory drugs (NSAIDs), immunosuppressants, aspiration or surgery [4]. A review of the literature suggests that topical and systemic corticosteroids are currently the most used treatment for IGM. The use of corticosteroids has been reported to have high efficiency, negligible recurrence rates, and a good aesthetic outcome, making it the current first line therapy [5]. Alternatively, the addition of immunosuppressants like methotrexate to corticosteroid therapy has also been reported to increase treatment success and allow faster tapering of steroid doses [6]. Non-targeted antibiotic treatment is another alternative, however, its lack of benefit has generally been accepted. Surgery is often a last resort due to its morbidity and high rates of post-surgical recurrence rates up to 50%. Several other alternative therapeutic options have also been explored, such as traditional Chinese herbal medicine [7], ultrasound-guided microwave ablation [8], ductal lavage [9], and even ozone therapy [10]. Although its etiology remains unknown, an autoimmune origin is the most favored underlying cause to date due to its established triggers and its response to corticosteroid and immunomodulator treatments [11].

Several features of IGM are linked to auto-immune diseases, and these features are the following: 1) erythema nodosum which is a typical systemic manifestation of IGM, 2) co-existence with other immune diseases such as SLE and Sjogren’s syndrome, and 3) seasonal occurrence of disease onset.[12] Known risk factors associated with the development of IGM that are related to auto-immune diseases include pregnancy and lactation, hormonal imbalance specifically hyperprolactinemia, trauma, smoking, and infection with Corynebacterium species being the most common infectious agent implicated [12]. Through these triggers, Benson and Dumitru [13] postulated that damage to ductal epithelial lining (possibly from retention of ductal secretions) would lead to efflux of the duct contents to the lobular connective tissue, leading to local inflammation and migration of lymphocytes and macrophages to ductal zones causing non-caseating granuloma. Overall, these theories support the position that IGM is an auto-immune condition.

The most common first line treatment has been oral steroids. However, despite its benefits, the current first choice therapy of corticosteroids poses downsides such as frequent recurrences on discontinuation of therapy and impaired quality of life [14]. Therefore, there is a need to find an alternative therapy that is generally more efficacious than corticosteroids. Many studies have described the use of methotrexate in combination with steroids at different stages in the disease. For example, some studies describe switching from steroids to methotrexate after a lack of response, presence of adverse reactions, or following a relapse of disease after the initial steroid monotherapy treatment. However, methotrexate monotherapy in the absence of steroids is rarely reported. The few studies that have involved methotrexate monotherapy have shown promising remission rates and low adverse reaction rates to the methotrexate [15]. Methotrexate use was found to be associated with a high complete response rate of up to 75% [16] and relapse rates are significantly lower than steroid therapy and surgical intervention [17]. Hence, considering the need for the development of an alternative treatment to steroid monotherapy, this trial seeks to explore the potential of methotrexate monotherapy as a therapeutic option in the treatment of IGM.

The primary objective of this pilot trial is to investigate the effectiveness of prednisolone versus methotrexate in the treatment of IGM over a period of one year, in terms of the patients’ clinical and radiological response. The key secondary objectives would be to identify the associated side effects of the drugs, the frequency of percutaneous or surgical intervention required, potential biomarkers that may predict the response to treatment, patient reported outcome measures, and to validate the survey instrument for IGM patients.

## Materials and methods

### Ethics and dissemination

This study received ethical approval from the National University Hospital Institutional Review Board (DSRB reference number 2023/00773), regulatory approval from the Health Sciences Authority (HSA, CTA2400084), and is registered on ClinicalTrials.gov under the identifier NCT06943482. Written informed consent will be obtained from the patient after a qualified study team member explains the study and the patient has fully understood the study. Full disclosure of the potential benefits, disadvantages, including adverse events that may happen involving the two treatments will be discussed as well. Research findings will be submitted to publishing platforms and subjected to peer-reviewed, or presented to stakeholders, or shared in local and international conferences, in line with the ethics requirements.

### Study design and setting

This is a prospectively registered, open-label, two arm randomised controlled trial that will be conducted at the National University Hospital Singapore among patients who will be diagnosed with idiopathic granulomatous mastitis.

### Recruitment and sample size calculation

The aim will be to recruit 40 IGM patients, aged between 21–60 years, from NUH. Patients must have undergone breast ultrasound, biopsy, and a histopathology compatible with IGM. Eligible patients will be informed by their attending physician about this trial during the clinic session. A study coordinator will give further details about the study and obtain informed consent to interested patient prior enrolment. Patients will be assigned a participant study code and to one of the two following treatment arms: (1) Control arm: Prednisolone and (2) Experimental arm: Methotrexate. 20 patients will be included under the control arm, and 20 patients will be included under the treatment arm.

The sample size was calculated based on the prevalence rate of IGM in NUH being 10–15 patients per year. Based on a stepped rule of thumb, a minimum sample size of 30 will provide at least 80% power to detect a medium standardised effect size of between 0.3 to 0.7 based on a randomised two-group comparison [18]. Given the exploratory nature of this trial, the rarity of the disease, and feasibility considerations, a medium standardized difference was chosen as a pragmatic balance between statistical rigor and recruitment feasibility. Assuming a 20% attrition rate, the sample size required for this study is 36 patients. This number is hence rounded up to a sample size of 40 patients over a period of 36 months. This study has not yet commenced; participant recruitment is expected to begin in the second quarter of 2025. Participant recruitment and data collection is anticipated to be completed by the first quarter of 2027, with results expected by the end of 2027.

### Inclusion criteria

The inclusion criteria for patients who are allowed to take part in this study are as follows:

- Women, aged between 21 and 60 years
- Positive diagnosis compatible with idiopathic granulomatous mastitis based on histopathology results
- Histopathology results must show non-caseating granulomas (supported by clinical manifestation and pathological feature)
- Willing and able to give informed consent

### Exclusion criteria

Participants who meet any of the following prerequisites will not be allowed to take part in this study:

- Women who are currently pregnant or breastfeeding
- Cognitive impairment which prevents the patient from giving voluntary consent
- History of any psychiatric conditions such as depression, psychosis, schizophrenia etc.
- History of cancer in the past 5 years
- History of abnormal renal or liver function
- Poorly controlled diabetes mellitus
- Hepatitis B and/or Hepatitis C carrier
- Diagnosed with tuberculosis (Positive microbiological evaluation for Grocott Methenamine Silver stain and Ziehl– Neelsen stain)
- Any immunosuppressants or anti-inflammatory medications such as NSAIDS for the past 3 months
- Concomitant medication that may have contraindication with prednisolone and methotrexate use

### Withdrawal criteria

Patients will be withdrawn from the study upon their request. However, data that has been collected until the time of withdrawal will be kept and analysed. In cases of persistent non-compliance to dosing or study procedures, the data on non-compliance will be recorded, and the outcomes will be analysed based on intention-to-treat. There will be no replacement for patients who withdraw during the treatment period.

Under the following circumstances, patients will continue to be retained in the study to collect outcome data, but will be discontinued from taking the study drug:

- Serious allergic reaction to study drug requiring medical intervention and/or hospitalisation and as decided by investigators for safety reasons
- Development of adverse drug reactions to the prescribed trial drugs which require medical intervention and as decided by investigators for safety reasons
- Needing ventilator support upon hospitalisation, which prevents the consumption of tablets orally

### Randomisation and blinding

Permuted block randomisation will be performed, with randomly varying block sizes of 4 and 6. Eligible participants who have already enrolled will then be randomly assigned to either of the treatment arm based on their baseline visit date.

### Participant timeline

**Fig 1:**
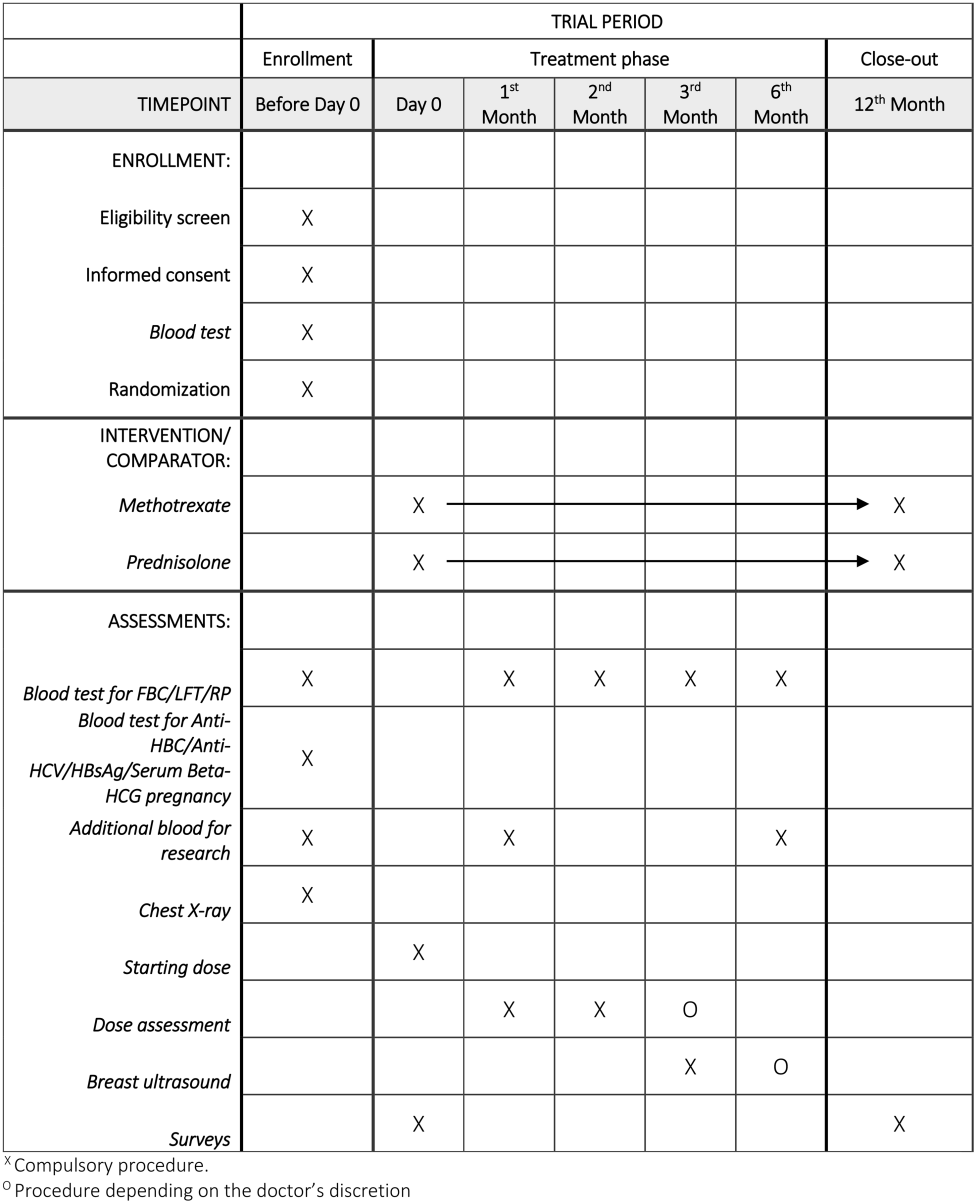
SPIRIT schedule of enrollment for prednisolone and methotrexate treatment arms.

The general timeline of the study consists of 3 main phases and is summarized in Fig 1: [19] the screening phase, the treatment phase, and the follow-up phase.

### Intervention

**Fig 2:**
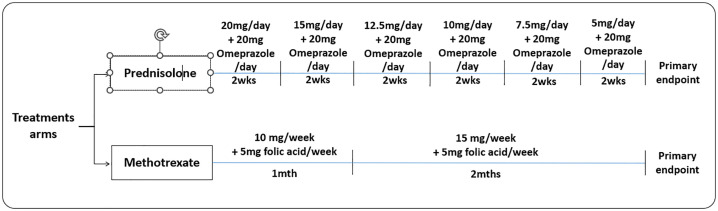
Flowchart of the treatment schedule for prednisolone and methotrexate during the treatment phase.

Patients in the control arm will receive 20 mg of prednisolone daily for first 2 weeks, followed by tapering doses of 15 mg daily over 2 weeks, then 12.5 mg daily for 2 weeks, proceeding to 10 mg daily for 2 weeks, subsequently taking 7.5 mg daily for 2 weeks and lastly 5 mg daily for 2 weeks (Fig 2). 20 mg of omeprazole is to be taken together with prednisolone. On the other hand, 10 mg of methotrexate will be prescribed to patients under experimental arm to be taken once a week for a month and increased to 15 mg of methotrexate to be taken once a week for another two months. 5 mg of folic acid is to be taken together with methotrexate once a week. This dosing schedule reflects the expected onset of methotrexate efficacy (≈2–6 weeks) and standard treat-to-target practice, initiating at a tolerable dose and escalating after 4 weeks if well tolerated to achieve a minimally effective weekly dose of 15 mg while limiting early toxicity. The 4-week review allows assessment of clinical response and laboratory safety before escalation. Folic acid 5 mg once weekly is included to reduce mucosal and hepatic adverse effects without compromising methotrexate efficacy [20]. For both arms, treatment will span 3 months, as this reflects the conventional duration of corticosteroid therapy for idiopathic granulomatous mastitis reported in prior studies and commonly practiced in clinical settings. A 3-month period provides sufficient time to evaluate treatment response while minimizing the risk of prolonged drug-related toxicity, ensuring both efficacy assessment and patient safety.

Drug dosage may be modified according to patient’s response to treatment and the attending’s clinical judgment.

### Specific restrictions related to safety

During the course of the study, patients on both treatment arms will be instructed to/advised:

1. Not get pregnant or try to get pregnant
2. No breastfeeding
3. Avoid alcohol
4. Inform their attending before getting any vaccinations during study period
5. Strictly refrain from concurrent dosing with other medicines, including prescriptions, and to inform the investigator(s) immediately about any new medication they need/want to take
6. Take medication as per packaging instructions

For patients on methotrexate, an additional instruction of not consuming tolbutamide and/or co-trimoxazole will be given, due to possible drug interactions.

#### Screening phase.

After informed consent, baseline blood test (32 ml, including pregnancy test, Hepatitis B test, Hepatitis C test, full blood count, liver function test and renal function test) and chest X-Ray will be conducted during the baseline visit to assess for eligibility to treatment phase. Additional research blood (20.5 ml) will be collected at this baseline visit for research purposes. Patient may be exempted from chest X-Ray and/or blood test if patient has done the same test within 30 days.

Baseline demographics and relevant clinical variables of patients will be collected: age at presentation, menopausal status, parity, breastfeeding status, concomitant rheumatic conditions, personal or family history of tuberculosis, smoking or alcohol intake, presenting complaint, unilateral or bilateral involvement, affected quadrant of the breast, duration of symptoms and previous antibiotics prescription for treatment of IGM.

Information on comorbidities will be evaluated to ensure suitability for both treatment arms prior to recruitment. This includes drug allergies, plans for pregnancy, ongoing infection, previous history of cancer, previous tuberculosis, epilepsy, diabetes, hypertension, kidney, liver, cardiac problems, osteoporosis, glaucoma, peptic ulcers, previous side effects to either prednisolone or methotrexate.

All eligible patients after screening assessment will be randomised to either of the treatment arm. Ineligible patients will not proceed to the treatment phase, however data collected and additional blood samples will be kept for analysis.

#### Treatment phase.

After screening eligibility and randomisation, patients will be scheduled to attend a clinical visit (T0) to either breast or rheumatology clinic for the initiation of treatment dose. There will be a window period of ± 7 days for this clinical visit. Patient is required to complete a pre-treatment quality of life online survey (approximately 10–15 minutes).

Patients will be scheduled for blood test (8mls, 0.5 tablespoons) and clinical visits to either breast or rheumatology clinic at the 1st (T1), 2nd (T2), 3rd (T3) and 6th (T4) month to assess their signs and symptoms, and drug compliance. There will be a window period of ± 14 days for each of these clinical visits. Additional research blood (20.5mls, 1.5 tablespoons) will be collected on the 1st (T1) and 6th (T4) months.

Patients will be required to undergo a breast ultrasound during the 3rd (T3) clinical visit to assess for their radiological response. A window period of ± 14 days will be applicable. The initial measurement of the breast lesion or mass will be recorded in three dimensions: anteroposterior, superior-inferior, mediolateral measurements. If complete clinical and radiological complete response has been achieved on the 3rd (T3) visit, the drug will be discontinued in a safe manner and no further breast ultrasounds will be required. However, if symptoms or signs have not resolved by the 3rd (T3) clinical visit, drug dosage will be assessed, and treatment may be continued up to the 6th (T4) month period and a repeat breast ultrasound will be required at the 6th (T4) month. If symptoms persist by the 6th (T4) month of clinical visit, the patient’s drug dosage may be up-titrated or will be given an addition of a second therapy, i.e., combination therapy of steroid and methotrexate. Clinical visits will coincide with standard care visit.

Throughout the course of the study, there are possible flares and relapse of the disease. Flare is defined as any increase in M-score or lesion size on ultrasound whilst on therapy, while relapse is defined as recurrence of symptoms or signs in the breast after previously experiencing complete resolution following therapy. In the event of flares, blood test (8mls, 0.5 tablespoons) and additional research blood (20.5mls, 1.5 tablespoons) will be collected and the allocated treatment for the patient will be up-titrated by the doctor. In event of relapse, treatment for the patient will be reinitiated as per the allocated arm for 6 months.

Additional research blood samples collected during the trial will be used for gene expressing profiling to identify unique patterns of gene expression that can be used to diagnose IGM and predict its treatment response.

Regardless of treatment arm, patient will be monitored by either breast surgeon or rheumatologist. Surgeons will assess for eligibility into trial, clinical reviews, and breast ultrasound interpretation. Rheumatologists will be monitoring for drug side effects and titration if required. Surgical intervention will be required in the event of failure of medical therapy. In the event where aspiration or surgical drainage is required, methotrexate or prednisolone should be continued unless there is evidence of sepsis. The frequency of aspiration or surgical intervention will be recorded as events during the study.

Whenever the disease has been resolved during the study period, the balance medication and/or packaging will be retrieved to do medication accounting and checking for adherence to assigned treatment arm.

If predefined, unexpected, severe adverse effects were to develop, doctors will evaluate their severity and withdraw the patients from the trial if needed, and these will be recorded as events as well.

#### Follow-up phase.

The last clinical visit at the 12th (T5) month may be a physical clinical visit or teleconsultation, depending on the doctor’s decision. Patient will be asked to complete a post-treatment quality of life online survey (approximately 10–15 minutes) during this visit. There will be a window period of ± 30 days for the last clinic visit.

### Outcome measures

#### Primary outcome.

The primary effect parameter is measured using Chen, et al’s “M-score”, [9] which ranges from 0 to 10, serving as a quantitative measurement of the severity of each patient’s symptoms. The scoring criteria is defined as the sum of the following scores:

1) Mass score: 0 for the absence of a mass by palpation, 1 for mass <3 cm by palpation, 2 for mass >3 cm by palpation
2) Erythema score: 0 and 2 for the absence and presence of skin erythema, respectively
3) Fistula score: 0 and 2 for the absence and presence of fistula, respectively.
4) Pain score: 0 for Visual Analogue Score (VAS) 0–2, 1 for VAS 3–5, 2 for VAS 6–10
5) Quality of life (QoL) score: 0 for the absence of effects on QoL, 1 for mild effects on QoL, where the patient does not require medical assistance, 2 for serious effects on QoL, where the patient requires medical assistance

The primary factor used to measure patients’ outcomes as a whole will be the complete clinical response (cCR) rate, which is defined as the proportion of patients who have an M-score of less than or equal to 1 within a year after treatment. Complete clinical response (cCR) is defined as reaching all of the criteria:

1) VAS 1;
2) Disappearance of all local symptoms, such as skin erythema, pain, and swelling;
3) Disappearance of fistula, if any;
4) The patient can return to normal life without any medical assistance; and
5) Disappearance of palpable mass

Alongside cCR, the researchers will also determine the time to cCR post randomization, treatment failure (TF) rate, and relapse rate (RR). Treatment failure is defined as either of the following: an M-score of ≥6 before randomisation which remained the same 1 month post randomisation; an M-score between 4 and 5 before randomisation which remained at ≥4 and never being lower than 4; and an M-score <4 at baseline but never reaches cCR after randomisation which remained >5 at the follow-up and remained above 5 for one month. Relapse moreso, is defined as an M-score of >4 among patients who achieved CR.

Radiological complete response will be defined as the complete resolution of all abnormal findings on breast ultrasound. This includes disappearance of any previously identified hypoechoic or heterogeneous mass, normalization of parenchymal architecture, and absence of perilesional inflammatory changes such as ductal dilatation, edema, or increased vascularity. Ultrasound assessments will be performed according to standardized breast imaging reporting and reviewed by radiologists experienced in breast imaging who will be blinded to treatment allocation to enhance consistency and reproducibility.

#### Secondary outcomes.

Secondary outcomes will include

1) Predefined and/or unexpected adverse effects between the two treatment arms will be recorded and compared. Predefined adverse events to both groups are the following:
  - Prednisolone may cause mood changes (confusion, depression), weight gain, fluid retention, nausea, vomiting, indigestion, easy bruising, blood or black, tarry tools, blurred vision, increased urination or thirst, muscle weakness or cramps and severe stomach pain.
  - Methotrexate may cause mouth ulcers, nausea, vomiting, abdominal pain, diarrhoea, rash, hair loss, easy bruising, severe sore throat, tiredness, paleness, yellow eyes, tea-coloured urine, constant abdominal pain, risk of infection and breathlessness or persistent dry cough.
2) Frequency of surgical intervention will also be recorded. Surgical intervention is defined as incision and drainage, wound debridement, excision, or mastectomy.
3) Biomarkers and gene profiling will also be determined which may predict treatment response.
4) Survey questionnaire will be administered to patients during the pre-treatment and follow-up period to measure patient reported outcomes.

### Adverse events and safety

Baseline laboratory tests and procedures at the baseline visit will be obtained to ensure that patients meet the inclusion and exclusion criteria. One of the safety precaution tests conducted would be a pregnancy test as drugs used in this trial may impair fetal development, if patient is pregnant. Methotrexate may occasionally lower blood count and cause liver inflammation. To detect these symptoms early, patients under the methotrexate arm will be required to undergo a blood test on the 1st (T1), 2nd (T2), 3rd (T3) and 6th (T4) month. If an increase in dosage in methotrexate is required by the doctor, patients will continue to undergo a blood test as per doctor’s discretion.

Both prednisolone and methotrexate are drugs that are widely used for treatments of other diseases and have an excellent safety profile. The study investigators will make every effort to mitigate risks and ensure that the inclusion and exclusion criteria are strictly adhered to. Study investigators will also conduct close monitoring on the patients’ well-being during the clinical visits. However, in the event of adverse events (AEs) and severe adverse events (SAEs), the principal investigator will be responsible for reporting to the ethics board (DSRB and HSA) in accordance with the reporting requirement.

An independent data monitoring committee composed of biostatistician and clinical experts will be established to monitor the study progress, compliance, and data governance. The committee will meet on regular basis and will be responsible for independently evaluation of the safety for the patients participating in the clinical trial.

### Data collection and management

In this trial, recruitment and follow-up survey data will be collected electronically via NUHS REDCap. The data will be routinely checked by study coordinators and study team members for the accuracy and completeness of the data. Only study team members with delegated rights will be able to access the data under the supervision of PI and Site-PI. All patient survey data will be collected electronically via FormSG for the pre-treatment and post-treatment online surveys. A copy of de-identified data will be stored in a secured server at NUH.

### Statistical and analytical plans

The primary outcome of 6-month clinical or radiological complete response will be compared between arms using Chi-square test. The effect estimate will be quantified in terms of difference in proportion as well as relative risk (RR) and their associated 95% confidence interval (CI). The secondary outcomes of time-to clinical or radiological response and time-to-relapse will be evaluated via the Kaplan-Meier method.

Differences in survival curves will be assessed with the log-rank test, and effect estimates expressed as hazard ratios (HR) with 95% CI. In addition, Cox proportional hazards models will be employed to account for potential imbalances in covariate distribution and to provide adjusted estimates of treatment effect. The differences in survival curves will be compared using log rank test, with the effect estimate quantified in terms of hazard ratio and its 95% CI. The primary analysis of this trial will be based on Intention-to- Treat (ITT). A Per Protocol (PP) analysis will be performed on all patients who have completed 6 months of treatment regimen as a sensitivity analysis. A sensitivity analysis will also be performed using the continuous M-score (without dichotomization), with group means compared between intervention and control arms using a two-sample t-test.

As this is a pilot RCT with a modest sample size, missingness will be minimised through active follow-up; remaining missing outcome data will be handled using multiple imputation by chained equations under a missing-at-random assumption. Complementary sensitivity analyses will include complete-case and best–worst/worst–best scenarios. For time-to-event outcomes, participants without events will be right-censored at last contact, with an additional analysis using inverse-probability-of-censoring weights if differential loss to follow-up is observed.

The z-test will be used to compare the proportions of side effects and the need for percutaneous or surgical intervention between treatment arms. Modified Poisson regression will be applied to identify clinical factors or biomarkers associated with treatment response and to quantify the effect in terms of relative risk.

Analyses will be conducted using R version 4.5.1 (R Foundation for Statistical Computing, Vienna, Austria), with a two-sided p-value of <0.05 considered statistically significant.

Patient reported outcomes will be reported via a survey on FormSG, conducted online. The questions will be adapted from SF36 V1 Singapore version and EORTC-BR23. Below are the domains to be compared: (1) General Health, (2) Physical Health, (3) Mental Health, (4) Body Image, (5) Sexual Image, and (6) Breast Health.

## Discussion

Many previous works, both prospective and retrospective, have explored treatment for IGM. From mere observation [21], to pharmaceutical treatment with steroids and immunosuppressants [22–30], to more invasive ones like ductal lavage [9], or combination of these modalities [31–33]. Although all treatment methods yielded appropriate efficacy and relapse prevention, corticosteroids more often resulted to unfavorable side effects and surgery causes significant disfigurement. Use of immunomodulators however promises lesser side effects with similar outcomes. Hence, this trial was contemplated to ultimately help patients gain treatment and remission without compromising safety and quality of life.

Oral corticosteroids remain one of the most commonly used treatment for IGM [22–28,34], hence it was chosen as the control arm. The dosage was based on Tan, et al’s study [35] which described that with oral 20 mg methylprednisolone monotherapy in IGM, 80.7% of patients responded well to steroid treatment. The rationale for a lower starting dose of oral prednisolone of 20 mg for Asians is due to higher risk of developing side effects to steroid as compared to their Caucasian counterparts. Omeprazole is to be taken concomitantly with prednisone to reduce gastric irritation brought about by it.

The use of methotrexate in the treatment of IGM is supported by the theory that IGM is an autoimmune disease [12]. The dosage was based on Postolva, et al’s study [16], with oral methotrexate monotherapy (starting dose of 10–15 mg/week and increased to 20–25 mg/week given orally or subcutaneously) in IGM, 94% had disease improvement. Only oral formulation of methotrexate was chosen in the study to standardize the bioavailability and ease of administration in this trial. Folic acid is to be taken concomitantly with methotrexate to reduce bone marrow suppression.

The study team believes that this is the first protocol to investigate steroid vs methotrexate treatment in a randomized trial setting. It will reverse the order of using methotrexate before steroids, focusing on methotrexate as a first-line treatment rather than a back-up management for IGM. This trial is the first of its kind to compare two separate arms for IGM treatment to inform breast physicians on the comparability of these arms. By doing so, the study could potentially reinvent the way IGM is treated and approached in women. Depending on the treatment outcomes of the trial, it may be possible to secure a first-line immunosuppressant protocol for IGM by acquiring a more robust understanding of the disease’s response to immunosuppressants and immunomodulators. This could lead to possibly establishing methotrexate as a novel treatment for IGM, or the exploration of the use of other immunomodulators in the future. Additionally, the study aims to develop a biomarker bank in Singapore which will allow prediction of patients’ response to novel treatment based on the relevant biomarkers. This will expand the current understanding of IGM’s etiology and allow physicians to develop and test new novel treatments for IGM in the future. The trial also involves inter-departmental collaboration, with a strong collaboration with the department of rheumatology. IGM might possibly be a disease of immunological etiology and hence, inter-departmental collaboration will bring together the knowledge and expertise from different disciplines into achieving a more comprehensive understanding of IGM.

Some limitations of the pilot trial that can be foreseen would be slow recruitment from a single center. Patients may also choose no treatment at all. A small sample size will reduce the trial’s statistical power and make it more challenging to detect true differences between the 2 arms. However, the primary aim is to generate preliminary results for future multicentre trial. Another foreseen issue is the possibility of observer bias due to lack of blinding. As much as the study team wants to eliminate such, blinding cannot be achieved in this study since the medications are taken at different schedules. Moreover, both drugs can pose significant side effects and hence patient safety is the study team’s utmost priority. Moving forward, this trial could potentially be the catalyst for further studies exploring the underlying pathophysiology of IGM, allowing a more comprehensive understanding of this complex disease. Physicians can also explore the use of other immunosuppressants/immunomodulators other than prednisolone or methotrexate for the treatment of IGM, and the efficacy of step-up or top-down approaches.

## Conclusion

To the best of the researchers’ knowledge, this study is the first to compare oral corticosteroid and methotrexate in a randomized control setting. Methotrexate is hypothesized to achieve higher clinical or radiological complete response, demonstrate a more favorable side effect profile, reduce the need for surgical intervention, and improve patient-reported outcomes. The goal is to deliver robust evidence for the treatment and management of IGM.

## Supporting information

S1 File Original study protocol. (DOCX)

S2 File Ethics approval letter. (PDF)

S3 File SPIRIT Fillable-checklist IGM. (PDF)
