# Supplementary material for: Protocol for a randomized controlled trial of steroid versus methotrexate as first-line monotherapy in the management of idiopathic granulomatous mastitis
Source: PLoS One. 2025 Oct 27;20(10):e0333577. doi: 10.1371/journal.pone.0333577 (PMC12558474; doi:10.1371/journal.pone.0333577)
Supplement: S1 File — (DOCX) [file pone.0333577.s001.docx]

so

**STUDY PROTOCOL**

| **PROTOCOL TITLE:** | |
| --- | --- |
| A pilot open-label randomised trial comparing the effectiveness of steroids and methotrexate for the treatment of idiopathic granulomatous mastitis | |
|  | |
| **PROTOCOL NUMBER:** | |
| IGM001 | |
|  | |
| **PROTOCOL VERSION:** | 2.0 |
| **PROTOCOL DATE:** | 27 June 2024 |
|  | |
| **PRINCIPAL INVESTIGATOR:** | |
| Dr Serene Goh Si Ning, Department of Surgery, National University Hospital | |
|  | |
| **STUDY SITE:** | |
| National University Hospital | |
|  | |

**TABLE OF CONTENTS**

[1. BACKGROUND AND RATIONALE 4](#_Toc147244740)

[1.1. General Introduction 4](#_Toc147244741)

[1.2. Rationale and Justification for the Study 5](#_Toc147244742)

[a. Rationale for Doses Selected 5](#_Toc147244743)

[b. Rationale for Study Population 6](#_Toc147244744)

[c. Rationale for Study Design 6](#_Toc147244745)

[2. HYPOTHESIS AND OBJECTIVES 6](#_Toc147244746)

[2.1. Hypothesis 6](#_Toc147244747)

[2.2. Primary Objectives 6](#_Toc147244748)

[2.3. Secondary Objectives 6](#_Toc147244749)

[2.4. Potential Risks and benefits 7](#_Toc147244750)

[a. End Points – Efficacy 7](#_Toc147244751)

[b. End Points – Safety 7](#_Toc147244752)

[3. STUDY POPULATION 7](#_Toc147244753)

[3.1. List the number of subjects to be enrolled 7](#_Toc147244754)

[3.2. Criteria for Recruitment 7](#_Toc147244755)

[3.3. Inclusion Criteria 8](#_Toc147244756)

[3.4. Exclusion Criteria 8](#_Toc147244757)

[3.5. Withdrawal Criteria 8](#_Toc147244758)

[3.6. Subject Replacement 9](#_Toc147244759)

[4. TRIAL SCHEDULE 9](#_Toc147244760)

[5. STUDY DESIGN 9](#_Toc147244761)

[5.1. Summary of Study Design 9](#_Toc147244762)

[6. METHODS AND ASSESSMENTS 10](#_Toc147244763)

[6.1. Randomisation and Blinding 10](#_Toc147244764)

[6.2. Contraception and Pregnancy Testing 10](#_Toc147244765)

[6.3. Study Visits and Procedures 10](#_Toc147244766)

[7. TRIAL MATERIALS 13](#_Toc147244767)

[7.1. Trial Product (s) 13](#_Toc147244768)

[7.2. Storage and Drug Accountability 14](#_Toc147244769)

[8. TREATMENT 14](#_Toc147244770)

[8.1. Rationale for Selection of Dose 14](#_Toc147244771)

[8.2. Study Drug Formulations 14](#_Toc147244772)

[8.3. Study Drug Administration 14](#_Toc147244773)

[8.4. Specific Restrictions / Requirements 14](#_Toc147244774)

[8.5. Blinding 15](#_Toc147244775)

[8.6. Concomitant therapy 15](#_Toc147244776)

[9. SAFETY MEASUREMENTS 15](#_Toc147244777)

[9.1. Definitions 15](#_Toc147244778)

[9.2. Collecting, Recording and Reporting of “Unanticipated Problems Involving Risk to Subjects or Others” – UPIRTSO events to the NHG Domain Specific Review Boards (DSRB) 15](#_Toc147244779)

[9.3. Collecting, Recording and Reporting of Serious Adverse Events (SAEs) to the Health Science Authority (HSA) 16](#_Toc147244780)

[9.4. Safety Monitoring Plan 17](#_Toc147244781)

[9.5. Complaint Handling 17](#_Toc147244782)

[10. DATA ANALYSIS 17](#_Toc147244783)

[10.1. Data Quality Assurance 17](#_Toc147244784)

[10.2. Data Entry and Storage 18](#_Toc147244785)

[11. SAMPLE SIZE AND STATISTICAL METHODS 18](#_Toc147244786)

[11.1. Determination of Sample Size 18](#_Toc147244787)

[11.2. Statistical and Analytical Plans 19](#_Toc147244788)

[12. ETHICAL CONSIDERATIONS 19](#_Toc147244789)

[12.1. Informed Consent 19](#_Toc147244790)

[12.2. IRB review 19](#_Toc147244791)

[12.3. Confidentiality of Data and Patient Records 20](#_Toc147244792)

[13. PUBLICATIONS 20](#_Toc147244793)

[14. RETENTION OF TRIAL DOCUMENTS 20](#_Toc147244794)

**STUDY PROTOCOL**

| **BACKGROUND AND RATIONALE** |  |
| --- | --- |
| Idiopathic granulomatous mastitis (IGM) is an inflammatory breast disease affecting mainly young to middle aged women in Asia^1^. The disease has a chronic recurrent course with multiple flares which inevitably affects the quality of life in these women who are usually in their reproductive years^2^. It is also a disfiguring illness with potential negative psychosocial impact on young women^3^. Its aetiology is poorly understood, and its treatment remains an enigma to breast physicians.  There is a myriad of therapeutic options for IGM such as antibiotics, steroids, non-steroidal anti-inflammatory drugs, immunosuppressants, aspiration or surgery^4^. However, there is currently no consensus on the ideal treatment to date. The lack of benefit from non-targeted antibiotic treatment has generally been accepted^3^. Surgery is commonly last resort due to the breast deformity from breast tissue volume loss, surgical scarring and despite these, the risk of recurrence persists postperatively^5^. Prednisolone is a common first-choice therapy however it is associated with multiple side-effects, frequent recurrences on discontinuation of therapy and may be associated with impaired quality of life^6^. Therefore, there is need to consider alternative treatment options with fewer adverse reactions and impact on quality of life.  Methotrexate is presently considered second-line therapy and appears to have fewer side-effects^7^. To date, there are a few reports describing the use of methotrexate at different stages in the disease. For example, switching of steroid therapy to methotrexate after a lack of response to corticosteroid treatment or following a relapse of disease on steroid therapy or used as combination with steroids^8^ during the initial treatment. However, methotrexate monotherapy or in combination with low doses of corticosteroids were rarely reported. Nonetheless, methotrexate use was found to be associated with a high complete response rate of up to 75%^9^ and relapse rates are significantly lower than prednisolone and surgeries^10^. |  |
| General Introduction |  |
| This research study is to determine the effectiveness of prednisolone and methotrexate for IGM patients over a period of one year, in terms of clinical and radiological response. Recruitment will be conducted in National University Hospital (NUH) for patients diagnosed with IGM.   1. **Prednisolone**   Prednisolone is a member of the corticosteroid drug class. The hormones known as corticosteroids are produced naturally by the body and support overall health. Prednisolone can help lessen swelling, redness, and itching as well as signs of inflammation. Additionally, prednisolone can be used to treat ailments like, asthma, allergies, eczema, psoriasis, lupus and rheumatoid arthritis. (<https://www.healthhub.sg/a-z/medications/482/prednisolone>) As IGM is an inflammatory breast disease, prednisolone is often used as a first-choice therapy. However, it is associated with multiple side-effects, frequent recurrences after discontinuation of therapy and may be associated with impaired quality of life.   1. **Methotrexate**   Methotrexate is a medication that lowers immune system hyperactivity. It is used to treat several autoimmune illnesses, including inflammatory myositis, juvenile idiopathic arthritis, rheumatoid arthritis, systemic lupus erythematosus (SLE), psoriasis, and psoriatic arthritis. Methotrexate functions as a disease-modifying anti-rheumatic medication (DMARD) in the treatment of arthritis, assisting in the reduction of pain, swelling, and long-term joint damage.  (<https://www.healthhub.sg/a-z/medications/178/methotrexate>)  Presently, methotrexate is considered second-line therapy and appears to have fewer side-effects. To date, there are a few reports describing the use of methotrexate at different stages in the disease. For example, switching of steroid therapy to methotrexate after a lack of response to corticosteroid treatment or adverse reactions, or following a relapse of disease on steroid therapy or used as combination with steroids during the initial treatment. However, methotrexate monotherapy or in combination with low doses of corticosteroids were rarely reported. Nonetheless, methotrexate use was found to be associated with a high complete response rate of up to 75% and relapse rates are significantly lower than prednisolone and surgeries. |  |
| Rationale and Justification for the Study |  |
| Rationale for Doses Selected |  |
| Tan et al described that with oral 20mg methylprednisolone monotherapy in IGM, 80.7% of patients responded well to steroid treatment. The rationale for a lower starting dose of oral prednisolone of 20mg for Asians is due to higher risk of developing side effects to steroid as compared to their Caucasian counterparts. For this study, 20 mg of prednisolone will be prescribed daily for first 2 weeks, followed by tapering doses of 15mg daily over 2 weeks, then 12.5 mg daily for 2 weeks, proceeding to 10 mg daily for 2 weeks, subsequently taking 7.5mg daily for 2 weeks and lastly 5mg daily for 2 weeks (refer to Figure 2). 20mg of omeprazole is to be taken together with prednisolone. (Refer to Section 8.6 on justification for omeprazole).  Postolva et al described that with oral methotrexate monotherapy (starting dose of 10-15mg/week and increased to 20-25mg/week given orally or subcutaneously) in IGM, 94% had disease improvement^16^. Only oral formulation of methotrexate was chosen in our study to standardize the bioavailability and ease of administration in this trial^17^. In this study, 10mg of methotrexate will be prescribed to be taken once a week for a month and increased to 15mg of methotrexate to be taken once a week for another two months. 5mg of folic acid is to be taken together with methotrexate once a week (Refer to Section 8.6 on justification for folic acid).  *Modification of doses*  Modification of doses may occur during the study period and this will be dependent on patient’s clinical response and the attending doctor’s judgement. Principal Investigator (PI) or Co-Investigators (Co-Is) may increase the methotrexate dosage from 15mg to 20mg and may increase the 5mg folic acid intake up to 6 times a week (except the day of taking methotrexate) to mitigate methotrexate’s side effect, if needed. |  |
| Rationale for Study Population |  |
| The reported incidence of IGM is around 2.4 per 100,000 women aged 20–40 years. The study team will be including women aged 21 to 60 years because IGM often affects women who are young to middle aged. In addition, any women below the age of 21 years are unable to give consent independently in Singapore. |  |
| Rationale for Study Design |  |
| This is a prospective pilot randomised trial to study the effectiveness of prednisolone versus methotrexate in achieving clinical or radiological complete response for women with IGM. The study will be conducted in NUH. This study evaluates the effectiveness of each drug in terms of clinical and/or radiological response, time to resolution as well as total number of flares over a period of one year follow-up. This study can potentially identify a more effective treatment with fewer side effects for women with IGM as well as personalize drug choices for patients who are at risk of frequent flares. |  |
| **HYPOTHESIS AND OBJECTIVES** |  |
| Hypothesis |  |
| The hypothesis is that methotrexate has a higher 6-month clinical or radiologically complete response with lesser side effects than prednisolone. The time to clinical or radiological response is shorter in the methotrexate arm compared to prednisolone arm. Relapse and time to first relapse is lower and longer in the methotrexate arm compared to prednisolone arm. |  |
| Primary Objectives |  |
| The primary objective is to evaluate the 1) Proportion of patients who achieved clinical or radiological complete response at 6 months 2) Time to clinical response and time to radiological response, and 3) Proportion of patients with relapse in one year within affected breast between each arm. |  |
| Secondary Objectives |  |
| The secondary objectives include evaluation of:   1. Side effects 2. Frequency of percutaneous or surgical intervention required 3. Potential biomarkers that may predict response to treatment: eg IL-6, CRP and neutrophil to lymphocyte ratio (NLR) 4. Patient reported outcome measures 5. Validate survey instrument for IGM patients |  |
| Potential Risks and benefits |  |
| End Points – Efficacy |  |
| Benefits expected for the patient are the total remission of IGM or reduced relapsed/flare of IGM. |  |
| End Points – Safety |  |
| The risk anticipated for the patients are:  1. All research techniques and medications have potential dangers and adverse effects. Blood collection may result in discomfort, bleeding, bruising, or swelling where the needle was inserted. Fainting may occur, and infections happen infrequently.  2. Allergic reactions can occur with any drug such as rash, itching etc.  3. Rarely, a severe and possibly life-threatening allergic reaction can occur. Symptoms of a severe reaction include: swelling of the face, difficulty breathing, or a sudden drop in blood pressure that may cause dizziness.  4. For the study drugs, there are some side effects that are unique to the drugs (prednisolone and methotrexate).  i) Prednisolone may cause mood changes (confusion, depression), weight gain, fluid retention, nausea, vomiting, indigestion, easy bruising, blood or black, tarry tools, blurred vision, increased urination or thirst, muscle weakness or cramps and severe stomach pain.  ii) Methotrexate may cause mouth ulcers, nausea, vomiting, abdominal pain, diarrhoea, rash, hair loss, easy bruising, severe sore throat, tiredness, paleness, yellow eyes, tea-coloured urine, constant abdominal pain, risk of infection and breathlessness or persistent dry cough. |  |
| **STUDY POPULATION** |  |
| List the number of subjects to be enrolled |  |
| 40 IGM patients, aged between 21 to 60 years will be recruited from NUH. |  |
| Criteria for Recruitment |  |
| Patients must have undergone biopsy and diagnosed with IGM. Eligible patients will be informed by their attending healthcare professional on the study during the clinic session. If patient is interest to join the study, a qualified study team member will then explain the study and obtain informed consent from the patient. Before beginning any study-related procedures, informed consent will be obtained. Patient will then be assigned a participant study code and randomised to a treatment arm. Recruitment will be done face-to-face in NUH at Breast Care Centre. |  |
| Inclusion Criteria |  |
| Patients will have to meet all of the following criteria below:   - Women, aged between 21 and 60 years - Positive diagnosis of idiopathic granulomatous mastitis based on histopathology results - Willing and able to give informed consent |  |
| Exclusion Criteria |  |
| Patients who meet any of the following prerequisites will not be allowed to take part in this study:   - Women who are currently pregnant or breastfeeding - Cognitive impairment which prevents the patient from giving voluntary consent - History of any psychiatric conditions such as depression, psychosis, schizophrenia etc. - History of cancer in the past 5 years - History of abnormal renal or liver function - History of diabetes mellitus - Hepatitis B and/or Hepatitis C carrier - Diagnosed with tuberculosis (Positive microbiological evaluation for Grocott Methenamine Silver stain and Ziehl– Neelsen stain) - Any immunosuppressants or anti-inflammatory medications such as NSAIDS for the past 3 months - Concomitant medication that may have contraindication with prednisolone and methotrexate use |  |
| Withdrawal Criteria |  |
| Patients will be withdrawn from the study upon their request. However, data that has been collected until the time of withdrawal will be kept and analysed.  In cases of persistent non-compliance to dosing or study procedures, the data on non-compliance will be recorded, and the outcomes will be analysed based on intention-to-treat.  Under the following circumstances, patients will continue to be retained in the study to collect outcome data, but will be discontinued from taking the study drug:   - Serious allergic reaction to study drug requiring medical intervention and/or hospitalisation and as decided by investigators for safety reasons - Development of adverse drug reactions to the prescribed trial drugs which require medical intervention and as decided by investigators for safety reasons - Needing ventilator support upon hospitalisation, which prevents the consumption of tablets orally |  |
| Subject Replacement |  |
| There will be no replacement for patients who withdraw during the treatment period. |  |
|  |  |
| **4. TRIAL SCHEDULE** |  |
| Figure 1 below shows the study timeline for prednisolone and methrotexate treatment arm.  Figure 2 shows the drug dosage and schedule.  **Figure 1: Study Timeline for Prednisolone and Methotrexate Treatment Arm**   \| **Prednisolone/Methotrexate** \| \| **Study period (1 year)** \| \| \| \| \| \| \| \| --- \| --- \| --- \| --- \| --- \| --- \| --- \| --- \| --- \| \| **Phases** \| \| **Screening** \| **Treatment phase** \| \| \| \| **Follow-up phase** \| \| \| **Time point** \| \| Baseline \| T_0_ \| T_1_ \| T_2_ \| T_3_ \| T_4_ \| T_5_ \| \| Day 0 \| 1^st^  Month \| 2^nd^  Month \| 3^rd^  Month \| 6^th^  Month \| 12^th^  month \| \| Assessment \| Window Period (Day) \|  \| ±7 \| ±14 \| ±14 \| ±14 \| ±14 \| ±30 \| \| Clinical Visit \| X \| X \| X \| X \| X \| X \| X \| \| Blood Test for  FBC/LFT/RP \| X \|  \| X \| X \| X \| X \|  \| \| Blood Test for  Anti-HBc/Anti-HCV/HBsAg/ Serum Beta-HCG pregnancy test ^1^ \| X \|  \|  \|  \|  \|  \|  \| \| Additional Blood for Research \| X \|  \| X \|  \|  \| X \|  \| \| Chest X-Ray ^2^ \| X \|  \|  \|  \|  \|  \|  \| \| Starting Dose \|  \| X \|  \|  \|  \|  \|  \| \| Dose Assessment \|  \|  \| X \| X \| O \|  \|  \| \| Breast Ultrasound \|  \|  \|  \|  \| X \| O \|  \| \| Surveys \|  \| X \|  \|  \|  \|  \| X \|   X= compulsory procedure O=procedure depending on the doctor’s discretion   1. Blood tests (Anti-HBc/Anti-HCV/HBsAg) will be exempted if patient has done the same test within 30 days. 2. Chest X-Ray will be exempted if patient has done the same test within 30 days.   **Figure 2: Flowchart of the treatment schedule for prednisolone and methotrexate during the treatment phase.**  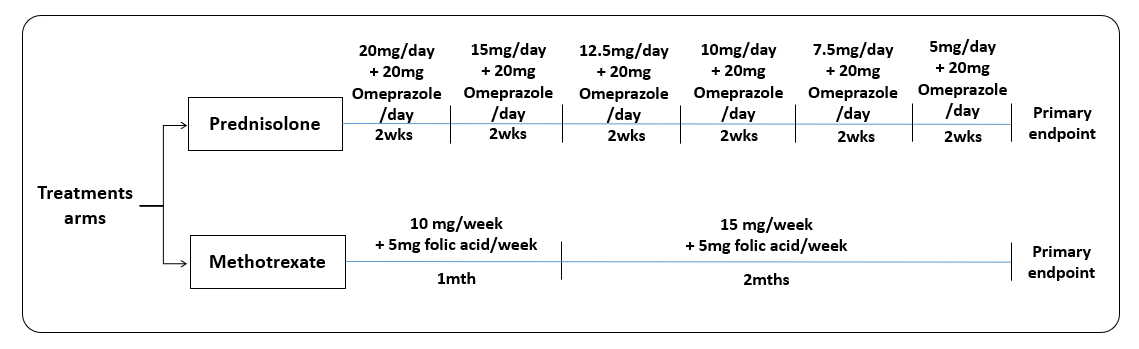 |  |
| **STUDY DESIGN** |  |
| Summary of Study Design |  |
| This is a prospective pilot randomised trial to study the effectiveness of prednisolone versus methotrexate in achieving clinical or radiological complete response for women with IGM. Patient will be recruited from NUH and written informed consent will be obtained prior to screening and enrolment. Patient will be randomised to one of the treatment arms.  The 2 arms consist of:   - Control arm (20 patients):   - Prednisolone tablet will start at 20mg daily for first 2 weeks (Refer to Figure 2 for tapering doses) with 20mg of omeprazole daily (Refer to Section 8.6) - Experimental arm (20 patients):   - Methotrexate tablet will start at 10mg once a week for the first month (Refer to Figure 2 for increasing of dosage) with 5mg of folic acid (Refer to Section 8.6) |  |
|  |  |
| **METHODS AND ASSESSMENTS** |  |
| Randomisation and Blinding |  |
| Permuted block randomisation will be performed with randomly varying block sizes of 4 and 6. Eligible and consented participants will then be randomly assigned to either of the treatment arm based on their baseline visit date. |  |
| Contraception and Pregnancy Testing |  |
| We do not accept women who self-report or test as pregnant during recruitment. |  |
| Study Visits and Procedures |  |
| **Study Visits and Procedures** |  |
| *Screening*  After patient is consented to the study, a baseline blood test (32mls, including pregnancy test, Hepatitis B test, Hepatitis C test, full blood count, liver function test and renal function test) and chest X-Ray will be conducted during the baseline visit to assess for eligibility prior to enrolment to the treatment phase. Additional research blood (14.5mls, 1 tablespoons) will be collected at this baseline visit for research purposes. Patient may be exempted from chest X-Ray and/or blood test if patient has done the same test within 30 days.  Baseline demographics and relevant clinical variables of patients will be collected. The following demographic and clinical variables will be collected: age of presentation, pre-menopausal or post-menopausal status, parity / breastfeeding status, concomitant rheumatic conditions, personal or family history of tuberculosis, smoking or alcohol intake, presenting complaint, unilateral or bilateral involvement, affected quadrant of the breast, duration of symptoms and previous antibiotics prescription for treatment of IGM.  Information on comorbidities will be evaluated to ensure suitability for both treatment arms prior to recruitment. Examples include drug allergies, plans for pregnancy, ongoing infection, previous history of cancer, previous tuberculosis, epilepsy, diabetes, hypertension, kidney, liver, cardiac problems, osteoporosis, glaucoma, peptic ulcers, previous side effects to either prednisolone or methotrexate.  If patient fulfilled all the eligibility criteria after screening assessment, she will be randomised to either of the treatment arm.  If patient did not meet the full eligibility criteria (eg. Pregnant, hepatitis carrier or abnormalities in renal or liver panels), she will not proceed to the treatment phase. The data collected and additional blood samples will be kept for analysis.  *Treatment Phase*  Patient will be scheduled to attend a clinical visit (T_0_) to either breast or rheumatology clinic if she meets all eligibility criteria for the initiation of treatment dose. There will be a window period of ± 7 days for this clinical visit. Patient is required to complete a pre-treatment quality of life online survey (approximately 10-15 minutes).  Patient will be scheduled for blood test (8mls, 0.5 tablespoons) and clinical visits to either breast or rheumatology clinic at the 1^st^ (T_1_), 2^nd^ (T_2_), 3^rd^ (T_3_) and 6^th^ (T_4_) month to assess their signs and symptoms, and drug compliance. There will be a window period of ± 14 days for each of these clinical visits. Additional research blood (14.5mls, 1 tablespoons) will be collected on the 1^st^ (T_1_) and 6^th^ (T_4_) months.  Patients are required to undergo a breast ultrasound to assess for their radiological response during the 3^rd^ (T_3_) clinical visit. A window period of ± 14 days will be applicable. The initial measurement of the breast lesion or mass will be recorded in three dimensions – i.e. anteroposterior, superior-inferior, mediolateral measurements. If complete clinical and radiological complete response has been achieved on the 3^rd^ (T_3_) visit, the doctor will discontinue the drug in a safe manner and no further breast ultrasounds will be required. If the patient’s symptoms or signs have not resolved by the 3^rd^ (T_3_) clinical visit, patient’s drug dosage will be assessed by the rheumatologist and treatment may be continued up to the 6^th^ (T_4_) month period and a repeat breast ultrasound will be required at the 6^th^ (T_4_) month. If symptoms have not resolved by the 6^th^ (T_4_) month of clinical visit, the patient’s drug dosage may be up-titrated or will be given an addition of a second therapy, i.e. combination therapy of steroid and methotrexate may be used. For clinical visits mention above, it will coincides with standard care visit.  During the clinical visits, Tingting Hu et al’s “M- score” will be adopted to grade the severity of the disease. M score is being calculated during clinical visits for IGM patients regardless of trial status for objective follow up. This is a common practice. For this study, the score will be administered by their attending healthcare professional (not limited to breast surgeons, investigators, or breast care nurses).  The M-score will be the sum of the following:  1) Mass score  2) Erythema score  3) Fistula score  4) Pain score  5) Quality-of-life (QoL) score  The scoring criteria were defined as follows:  1) Mass score: 0 for the absence of mass by palpation; 1 for mass ≤3 cm by palpation; 2 for mass >3 cm by palpation.  2) Erythema score: 0 and 2 for the absence and presence of erythema, respectively  3) Fistula: 0 and 2 for the absence and presence of fistula, respectively  4) Pain Score: 0 for Visual Analogue Score (VAS) 0-2; 1 for VAS 3-5; 2 for VAS 6-10  5) QoL score: 0 for absence of effects on QoL; 1 for mild effects on QoL, where the patient does not require medical assistant; 2 for serious effects on QoL, where the patient requires medical assistant  The total M-score ranges between 0 and 10 and serves as a quantitative and objective measurement of the severity of the symptoms.  The primary endpoint will be the clinical complete response (cCR) rate, which is defined as the proportion of patients who have an M-score of ≤ 1 at ≤1 years after treatment.  The secondary endpoints will be as follows:   - - The time to cCR, defined as the median time to cCR post randomisation.   - The treatment failure (TF) rate, defined as the proportion of patients with TF at 1 year post randomisation. The TF status will be defined as follows:     - If the patient has an M-score ≥6 before randomisation, TF is defined as the M-score remaining at ≥6 at 1 month post randomisation.     - If the patient has an M-score between 4 and 5 before randomisation, TF is defined as the M-score remaining at ≥4 and never being lower than 4.     - If the patient has an M-score <4 at baseline but never reaches cCR after randomisation, TF is defined as the M-score being >5 at the follow-up and remaining above 5 for one month. - Relapse rate, which is defined as the proportion of patients who have M-score >4 among the patients who achieved CR.   Throughout the course of the study, there are possible flares and relapse of the disease. Flares will be defined as any increase in M-score or lesion size on ultrasound whilst on therapy. While relapse will be defined as recurrence of symptoms or signs of IGM in the breast after previously experiencing complete resolution following therapy. If relapse occurs, patient will restart the same treatment arm for 6 months.  In the event of flares, blood test (8mls, 0.5 tablespoons) and additional research blood (14.5mls, 1 tablespoons) will be collected and the allocated treatment for the patient will be up-titrated by the doctor. In event of relapse, treatment for the patient will be reinitiated as per the allocated arm.  Additional research blood samples collected during the trial will be used for gene expressing profiling to identify unique patterns of gene expression that can be used to diagnose IGM and predict its treatment response.  Regardless of treatment arm, patient will be monitored by either breast surgeon or rheumatologist. Surgeons will assess for eligibility into trial, clinical reviews, and ultrasound breasts interpretation. Rheumatologists will be monitoring for drug side effects and titration if required. In event of failure of medical therapy, surgeons will have to intervene accordingly for aspiration or surgical drainage. In the event where patients require aspiration or surgical drainage during the study, methotrexate or prednisolone should be continued unless there is evidence of sepsis. The frequency of aspiration or surgical intervention will be recorded as events during the study.  Whenever the disease has been resolved during the study period, the balance medication and/or packaging will be retrieved to do medication accounting and checking for adherence to assigned treatment arm.  If side effects were to develop, doctors will evaluate the severity of the side effects and withdraw the patients from the trial if needed. |  |
| *Follow up Phase*  The last clinical visit at the 12^th^ (T_5_) month may be a physical clinical visit or teleconsultation, depending on the doctor’s decision. Patient will be asked to complete a post-treatment quality of life online survey (approximately 10-15 minutes) during this visit. There will be a window period of ± 30 days for the last clinic visit. Please refer to Figure 1 for the study timeline. |  |
| **iii. Post Study Follow up and Procedures** |  |
| If there are any unresolved adverse events that require medical intervention, follow-up visits will be conducted and verified through the electronic medical records of their hospitalisation.  In the case of serious adverse events, this will be reported to the DSRB as soon as possible, no later than 7 calendar days after first knowledge by the investigator, and any additional relevant information about the event should be reported within 8 calendar days of making the initial report.  Patients’ clinical information and personal data may be combined or linked with, including but not limited to: government administrative data and research data such as, nation-wide health-related databases, economic data and National Autoimmune Registry, up to 31 December 2043. The linkage may be carried out by itself or with the assistance of a data intermediary. The collection of such data is for the purposes of the current research study and will be collected prospectively. |  |
| **iv. Discontinuation Visit and Procedures** |  |
| A discontinuation visit will be conducted for all patients who discontinued the study drug or withdrawn from the study regardless of whether it is voluntary based on patient’s request or decided by the Investigators for safety or non-compliance issues to follow-up on:   - Balance medication and packaging retrieval to do medication accounting and checking for adherence to assigned treatment arm - Existing AE(s) /SAE or complaints that are not resolved and to ensure that care assessment with support care delivery is continued until the problem is resolved. - Reason for withdrawal (if requested by the patient) |  |
| **TRIAL MATERIALS** |  |
| Trial Product (s) The investigational product (IP) and Auxiliary Product (AP) will be procured locally from NUH pharmacy and stored at NUHS Investigational Medicine (IMU) facility. |  |
| Investigational Product (IP):   1. Prednisolone: 20mg/day. Y.S.P Prednisolone 5mg by Y.S.P. Industries (M) Sdn Bhd 2. Methotrexate: 15mg/week. Emthexate 2.5mg by TEVA Czech Industries S.R.O   Auxiliary Product (AP):   1. Omeprazole: 20mg/day. Zenpro Omeprazole 20mg by XEPA-SOUL Pattinson (Malaysia) Sdn Bhd 2. Folic acid: 5mg/day. Folic Acid Sunward tab 5mg by Sunward Pharmaceutical Pte Ltd |  |
| Storage and Drug Accountability |  |
| The PI and study team will be responsible to maintain the storage in IMU and drug accountability for all the trial product. | |
|  | |
| **TREATMENT** | |
| Rationale for Selection of Dose | |
| For safety reason, the dose of drugs used in this study is standard dosage in accordance with the approved labels. | |
| Study Drug Formulations | |
| All drugs dispensed to the patients are in tablet (methotrexate, folic acid, prednisolone) form and capsule form (omeprazole) for oral consumption. | |
| Study Drug Administration |  |
| 1. 20 mg of prednisolone daily for first 2 weeks, followed by tapering doses of 15mg daily over 2 weeks, then 12.5 mg daily for 2 weeks, proceeding to 10 mg daily for 2 weeks, subsequently taking 7.5mg daily for 2 weeks, and lastly 5mg daily for 2 weeks (refer to Figure 2). 20mg of omeprazole is to be taken together with prednisolone. 2. 15mg of methotrexate to be taken once a week for a month, and increased to 20mg of methotrexate to be taken once a week for another two months. 5mg of folic acid is to be taken together with methotrexate.   PI or Co-Is may increase the methotrexate dosage from 20mg to 25mg depending on patients’ responses to the drug and doctor’s discretion. PI or Co-Is may increase 5mg folic acid intake up to 6 times a week (except the day of taking methotrexate) to mitigate methotrexate’s side effects, if needed. |  |
| Specific Restrictions / Requirements |  |
| During the course of the study, patients on both treatment arms will be instructed to/advised:   1. Not get pregnant or try to get pregnant 2. No breastfeeding 3. Avoid alcohol 4. Inform the PI/Site PI/doctor before getting any vaccinations during study period 5. Strictly refrain from concurrent dosing with other medicines, including prescriptions, and to inform the investigator(s) immediately about any new medication they need/want to take 6. Take medication as per packaging instructions   For patients on methotrexate, an additional instruction of not consuming tolbutamide and/or co-trimoxazole will be given, due to possible drug interactions. |  |
| Blinding |  |
| This is a prospective pilot open label randomised trial. Patients and study team will not be blinded with regards to the treatment assigned. |  |
| Concomitant therapy |  |
| 20mg/day of omeprazole tablet will be taken with prednisolone tablet to reduce the irritation to the stomach caused by prednisolone.  5mg of folic acid tablet will be taken with methotrexate tablet every week to reduce bone marrow suppression. |  |
| **SAFETY MEASUREMENTS** |  |
| Definitions |  |
| Prednisolone and methotrexate have been widely used in local clinics for various diseases.    Prednisolone can be used to help reduce inflammation and reduce symptoms of swelling, redness and itchiness. It has been widely used to treat various diseases such as asthma, allergic conditions, skin problems, etc. It is also the standard of care treatment given to IGM patients in NUH and has a good safety profile.  Methotrexate is commonly used by rheumatologists for treatment of autoimmune diseases such as rheumatoid arthritis, systemic lupus erythematosus (SLE), etc.  Adverse events will be reported according to Common Terminology Criteria for Adverse Events (CTCAE) Version 5.0. |  |
| Collecting, Recording and Reporting of “Unanticipated Problems Involving Risk to Subjects or Others” – UPIRTSO events to the NHG Domain Specific Review Boards (DSRB) |  |
| **UPIRTSO events** refers to problems, in general, to include any incident, experience, or outcome (including adverse events) that meets ALL of the following criteria:   1. **Unexpected** 2. Pregnancy   In the event that the patient becomes pregnant during the study period, although very unlikely (patient will be screened for pregnancy before treatment phase and checked for pregnancy status at each clinical visit), patient will be withdrawn from the trial immediately. However, patient will be advised by their doctor or study investigator(s) on the procedures to stop medication.   1. Cancer   In the event that the patient is diagnosed with cancer during the study period, patient will be withdrawn from the trial immediately. However, patient will be advised by their doctor or study investigator(s) on the procedures to stop medication.   1. Fall risks   In the event that the patient falls during the treatment phase due to the side effect, doctor or study investigator(s) will assess and evaluate the severity of the fall before advising on the continuation of the study drug.  Expedited reporting to the IRB will be done as soon as possible, and not later than 7 calendar days after first knowledge by study investigator(s).   1. **Related or possibly related to participation in the research**   Prednisolone may cause mood changes (confusion, depression), weight gain, fluid retention, nausea, vomiting, indigestion, easy bruising, blood or black, tarry tools, blurred vision, increased urination or thirst, muscle weakness or cramps and severe stomach pain.  Methotrexate may cause mouth ulcers, nausea, vomiting, abdominal pain, diarrhoea, rash, hair loss, easy bruising, severe sore throat, tiredness, paleness, yellow eyes, tea-coloured urine, constant abdominal pain, risk of infection and breathlessness or persistent dry cough.   1. **Risk of harm**   Both prednisolone and methotrexate have been widely used clinically for treatments of other diseases and have a good safety profile.  The study investigators will make every effort to mitigate risks and ensure that the inclusion and exclusion criteria are strictly adhered to. Study investigators will also conduct close monitoring on the patients’ well-being during the clinical visits.  **Reporting Timeline for UPIRTSO Events to the NHG DSRB.**   1. Urgent Reporting: All SAE(s) listed whether related to or not to the study will be reported to IRB within 24 hours after first knowledge by study investigator(s):  - Results in death. - Is life-threatening (immediate risk of death). - Requires inpatient hospitalization or prolongation of existing hospitalization. - Results in persistent or significant disability/incapacity. - Is a medically important event.  1. Expedited Reporting: All collected study AE(s) will be reported as soon as possible, but not later than 7 calendar days after first knowledge by the study investigator(s). |  |
| Collecting, Recording and Reporting of Serious Adverse Events (SAEs) to the Health Science Authority (HSA) |  |
| A serious adverse event or serious adverse drug reaction is any untoward medical occurrence at any dose that:   - Results in death. - Is life-threatening (immediate risk of death). - Requires inpatient hospitalization or prolongation of existing hospitalization. - Results in persistent or significant disability/incapacity. - Results in congenital anomaly/birth defect - Is a medically important event.   All SAEs that are unexpected and related to the study drug will be reported. The investigator is responsible for informing HSA no later than 15 calendar days after first knowledge that the case qualifies for expedited reporting. Follow-information will be actively sought and submitted as it becomes available. For fatal or life-threatening cases, HSA will be notified as soon as possible but no later than 7 calendar days after first knowledge that a case qualifies, followed by a complete report within 8 additional calendar days. |  |
| Safety Monitoring Plan |  |
| Patients who consented to the study would have to undergo a few laboratory tests and procedures at the baseline visit to ensure that they meet the inclusion and exclusion criteria. One of the safety precaution tests conducted would be a pregnancy test to ensure that patient is not pregnant before consumption of drugs from either arm. The drugs used in this trial may impair fetal development, if patient is pregnant.  Methotrexate may occasionally lower blood count and cause liver inflammation. To detect these symptoms early, patients under the methotrexate arm is required to undergo a blood test on the 1^st^ (T_1_), 2^nd^ (T_2_), 3^rd^ (T_3_) and 6^th^ (T_4_) month. If an increase in dosage in methotrexate is required by the doctor, patients will continue to undergo a blood test as per doctor’s request.  An independent data monitoring committee which composes of biostatistician and clinical experts will be established to monitor the study progress, compliance, and data governance. The committee will meet on regular basis and will be responsible for independently evaluation of the safety for the patients participating in the clinical trial. Recommendation and concerns will be made by the committee for the study conduct and enrolment. In addition, the committee will regularly review the study Adverse Events (AEs) and Serious Adverse Events (SAEs). Investigator will be responsible to report relevant AE and SAE to the ethic board (DSRB and HSA) in accordance with the reporting requirement. |  |
| Complaint Handling |  |
| On the Informed Consent Form, there will be a contact number given that patient can raise their concerns to. A team will be designated to address the compliant, if any. All complaints will be kept anonymous. |  |
|  |  |
| **DATA ANALYSIS** |  |
| Data Quality Assurance |  |
| NUHS REDCap will be used to capture recruitment and relevant clinical data. The data will be routinely checked by study coordinators and study team members for accuracy and completeness of data. This IT system complies with applicable institutional data security, privacy, confidentiality, integrity and quality at NUHS cluster. FormSG will be used for the pre-treatment and post-treatment online surveys to capture patient related outcome measures. |  |
| Data Entry and Storage |  |
| De-identified blood samples will be analysed and stored in NUH Tissue Repository. Recruitment and follow-up survey data will be collected electronically via NUHS REDCap. Only study team members with delegated rights will be able to access the data under the supervision of PI and Site-PI. All survey data will be collected electronically via FormSG. A copy of de-identified data will be stored in a secured server at NUH. |  |
|  |  |
| **SAMPLE SIZE AND STATISTICAL METHODS** |  |
| Determination of Sample Size |  |
| The prevalence rate of IGM in NUH is currently at 10 to 15 patients per year. Based on a stepped rule of thumb, a minimum sample size of 30 will provide at least 80% power to detect a medium standardised effect size of between 0.3 to 0.7 based on a randomised two-group comparison (Machin et al, 2021). Assuming a 20% attrition rate, the sample size required for this study is 36 patients. This will be rounded up to a sample size of 40 patients over a period of 36 months. |  |
| Statistical and Analytical Plans The primary outcome of 6-month clinical or radiological complete response will be compared between arms using Chi-square test. The effect estimate will be quantified in terms of difference in proportion as well as relative risk (RR) and their associated 95% confidence interval (CI). The secondary outcomes of time-to clinical or radiological response and time-to-relapse will be evaluated via the Kaplan-Meier method. The differences in survival curves will be compared using log rank test, with the effect estimate quantified in terms of hazard ratio and its 95% CI. The primary analysis of this trial will be based on Intention-to- Treat (ITT). A Per Protocol (PP) analysis will be performed on all patients who have completed 6 months of treatment regimen as a sensitivity analysis.  Chi square test will be used to evaluate the proportion of side effects and requirement for percutaneous or surgical intervention during treatment between arms. Modified Poisson regression will be used to identify clinical or biomarkers that are associated with treatment response and quantify the effect in terms of RR.  Patient reported outcomes will be reported via a survey on FormSG, conducted online. The questions will be adapted from SF36 V1 Singapore version and EORTC-BR23. Below are the domains to be compared:   - - General Health   - Physical Health   - Mental Health   - Body Image   - Sexual Image   - Breast Health |  |
| **ETHICAL CONSIDERATIONS** |  |
| Informed Consent |  |
| Informed consent will be obtained from the patient after a qualified study team member explains the study and the patient has fully understood the study. The informed consent form is in a written format and will be explained orally by the qualified study team member to the potential patient in English with the presence of a witness. A translator will be present if the patient does not understand English. In addition, the translated short consent form will be used based on the patients’ preferred language. Sufficient time for the patients to review and consider participating will be given. "Your participation in this study is voluntary. You may stop participating in this study at any time. Your decision not to take part in this study or to stop your participation will not affect your medical care or any benefits to which you are entitled." is stated on the Patient Information Sheet and will be explained to patients. |  |
| IRB review |  |
| NUH has oversight purview over this study and will submit the protocol and the associated informed consent documents for the review and approval by the NHG DSRB.  This study will be conducted in accordance with the ethical principles that have their origin in the Declaration of Helsinki and that are consistent with the Good Clinical Practice and the applicable regulatory requirements.  This final study protocol, including the final version of the Patient Information Sheet and Informed Consent Form, must be approved in writing by the Cluster Ethic Review Board: DSRB prior to enrolment of any patient into the study.  The PI is responsible for informing the Cluster Ethic Review Board: DSRB of any amendments to the protocol or other study-related documents, as per local requirement. |  |
| Confidentiality of Data and Patient Records |  |
| The Principal Investigator and study team will be fully trained and will conduct the project strictly based on data protection and compliance policy according to institutions’ and ethic regulation. The patient records and de-identified blood samples will be separately coded and stored for maintaining patients’ confidentiality. All files are password protected and stored on secured servers with limited access.  However, there is still a potential risk of data breaching as data collection is involved. |  |
|  |  |
| **PUBLICATIONS** |  |
| Research findings will be submitted to publishing platforms and subjected to peer-reviewed, or presented to stakeholders, or shared in local and international conferences, in line with the ethics requirements. |  |
|  |  |
| **RETENTION OF TRIAL DOCUMENTS** |  |
| All source documents and IRB records from all participating sites will be kept securely in a locked cabinet in NUH with limited access and retained by overall PI for a period of 6 years after study completion, in accordance with the NUHS guidelines. If patient had consented for future research, their source documents will be stored for no longer than 20 years after study completion. |  |

REFERENCES

1. Altintoprak, F., Kivilcim, T., & Ozkan, O. V. (2014). Aetiology of idiopathic granulomatous mastitis. World journal of clinical cases, 2(12), 852–858. <https://doi.org/10.12998/wjcc.v2.i12.852>
2. Manogna, P., Dev, B., Joseph, L.D. et al. (2020). Idiopathic granulomatous mastitis—our experience. Egypt J Radiol Nucl Med, 51, 15. <https://doi.org/10.1186/s43055-019-0126-4>
3. Kehribar, D. Y., Duran, T. I., Cetinkaya, G. K., Polat, A. K., Ozgen, M. (2020). Evaluation of Symptoms, Depression and Anxiety Levels in Young Women with Idiopathic Granulomatous Mastitis. Int J Acad Med Pharm, 2(2), 57-61. <https://doi.org/10.29228/jamp.42734>
4. Steuer, A.B., Stern, M.J., Cobos, G., et al. (2020). Clinical Characteristics and Medical Management of Idiopathic Granulomatous Mastitis. JAMA Dermatol, 156(4):460–464. <https://doi.org/10.1001/jamadermatol.2019.4516>
5. Al Awfi, M. M., & Al Rahbi, S. K. (2023). Idiopathic Granulomatous Mastitis: Six years of experience and the current evidence in literature. Sultan Qaboos University medical journal, 23(1), 36–41. <https://doi.org/10.18295/squmj.4.2022.030>
6. Kayahan, M., Kadioglu, H., & Muslumanoglu, M. (2012). Management of Patients with Granulomatous Mastitis: Analysis of 31 Cases. Breast care (Basel, Switzerland), 7(3), 226–230. <https://doi.org/10.1159/000337758>
7. Kafadar, M. T., Bahadır, M. V., & Girgin, S. (2021). Low-Dose Methotrexate Use in Idiopathic Granulomatous Mastitis: An Alternative Treatment Method. Breast care (Basel, Switzerland), 16(4), 402–407. <https://doi.org/10.1159/000513879>
8. Kim, J., Tymms, K. E., & Buckingham, J. M. (2003). Methotrexate in the management of granulomatous mastitis. ANZ journal of surgery, 73(4), 247–249. <https://doi.org/10.1046/j.1445-1433.2002.02564.x>
9. Postolova, A., Troxell, M. L., Wapnir, I. L., & Genovese, M. C. (2020). Methotrexate in the Treatment of Idiopathic Granulomatous Mastitis. The Journal of rheumatology, 47(6), 924–927. <https://doi.org/10.3899/jrheum.181205>
10. Sheybani, F., Sarvghad, M., Naderi, H., & Gharib, M. (2015). Treatment for and clinical characteristics of granulomatous mastitis. Obstetrics and gynecology, 125(4), 801–807. <https://doi.org/10.1097/AOG.0000000000000734>
