# Supplementary material for: Protocol for a randomized controlled trial of steroid versus methotrexate as first-line monotherapy in the management of idiopathic granulomatous mastitis
Source: PLoS One. 2025 Oct 27;20(10):e0333577. doi: 10.1371/journal.pone.0333577 (PMC12558474; doi:10.1371/journal.pone.0333577)
Supplement: S2 File — (PDF) [file pone.0333577.s002.pdf]

National Healthcare Group
Adding years of healthy life

3 Fusionopolis Link
#03-08 Nexus@one-north
Singapore 138543
Tel: 6496 6600 Fax:6486 6870
www.nhg.com.sg
RCB No. 200002150H

NHG DSRB Ref: **2023/00773**

09 May 2024

Dr Serene Goh

Department of Surgery

National University Hospital

Dear Dr Goh

NHG DOMAIN SPECIFIC REVIEW BOARD (DSRB) APPROVAL

STUDY TITLE: A pilot open-label randomised trial comparing the effectiveness of steroids and methotrexate for the treatment of idiopathic granulomatous mastitis

We are pleased to inform you that the NHG Domain Specific Review Board has approved the application as titled above to be conducted in **National University Hospital**.

The approval period is from **09 May 2024** to **06 December 2024.** The NHG DSRB reference number for this study is **2023/00773.** Please use this reference number for all future correspondence.

Please note that this is a human biomedical research that is regulated by the Human Biomedical Research Act (HBRA) and researchers are required by law to comply with all the relevant regulatory requirements of the HBRA.

Where stipulated by the Research Institution, Principal Investigators and study team members must complete the HBR minimum training prior to commencement of study involvement.

Please note that clinical trials involving a Therapeutic and/or Medicinal Product can only be initiated after a Clinical Trial Authorisation/Notification/Certificate (CTA/CTN/CTC) has been issued or the Health Sciences Authority has given a written notification that a CTA/CTN/CTC is not required.

The documents reviewed are:

a) NHG DSRB Application Form: **Version No. 1**
b) Study Protocol: Version 1.2 dated 01 March 2024
c) Prednisolone Information Sheet: Version 1.0 dated 09 October 2023
d) Folic Acid Information Sheet: Version 1.0 dated 09 October 2023
e) Methotrexate Information Sheet: Version 1.0 dated 09 October 2023
f) Omeprazole Information Sheet: Version 1.0 dated 09 October 2023
g) Baseline Visit: Version 1.0 dated 09 October 2023
h) Scheduled Visit (Prednisolone): Version 1.0 dated 09 October 2023
i) Scheduled Visit (Methotrexate): Version 1.0 dated 09 October 2023
j) Unscheduled Visit: Version 1.0 dated 09 October 2023
k) Pre-treatment Survey: Version 1.0 dated 09 October 2023
l) Post-treatment Survey: Version 1.0 dated 09 October 2023
m) Eligibility Checklist: Version 1.1 dated 26 December 2023
n) Optional Consent Form for the Donation of Leftover Biological Samples: Version 1.0 dated 01 March 2024
o) Informed Consent Form: Version 1.2 dated 01 March 2024

Continued approval is conditional upon your compliance with the following requirements:

1. Only the approved Informed Consent Form should be used. It must be signed by each subject prior to initiation of any protocol procedures. In addition, each subject should be given a copy of the signed consent form.

2. Translated Informed Consent Forms (fully translated and short consent forms) and other translated documents are not required to be submitted to DSRB. It is the responsibility of the Principal Investigator to ensure that the translations for any document are an accurate reflection of the original approved content and to maintain the certification/documentation of the translations.

3. No deviation from or changes to the study should be implemented without documented approval from the NHG DSRB, except where necessary to eliminate apparent immediate hazard(s) to the study subjects.

4. Any deviation from or changes to the study to eliminate an immediate hazard should be promptly reported to the NHG DSRB within seven calendar days.

5. Principal Investigators and study team members are responsible for adhering to institutional guidelines, where applicable.

6. Please note that for studies requiring CTA/CTN/CTC, apart from the approval from NHG DSRB, no deviation from, or changes of the Research Protocol and Informed Consent Form should be implemented without documented approval from the Health Sciences Authority unless otherwise advised by the Health Sciences Authority.

7. Please submit the following to the NHG DSRB:

   a. All Unanticipated Problems Involving Risk To Subjects Or Others (UPIRTSOs) must be reported to the NHG DSRB. For more than minimal risk studies, all problems involving local deaths **must be reported as soon as possible**, but not later than 7 calendar days after first knowledge by the Investigator, regardless of the causality and expectedness of the death event, and any additional relevant information about the death should be reported within 8 calendar days of making the initial report. For no more than minimal risk studies, only problems involving local deaths that are related or possibly related to the study **must be reported as soon as possible**, but not later than 7 calendar days after first knowledge by the Investigator, and any additional relevant information about the death should be reported within 8 calendar days of making the initial report. For problems which are life threatening, it **must be reported as soon as possible**, but not later than 7 calendar days after first knowledge by the investigator, and any additional relevant information about the problems should be reported within 8 calendar days of making the initial report. All other problems that fulfil the UPIRTSOs reporting criteria **must be reported as soon as possible** but not later than 15 calendar days after first knowledge by the Investigator.

   b. Report(s) on any new information that may adversely affect the safety of the subject or the conduct of the study.

   c. NHG DSRB Study Status Report Form – this is to be submitted 4 to 6 weeks prior to expiry of the approval period. The study cannot continue beyond **06 December 2024** until approval is renewed by the NHG DSRB.

   d. Study completion – this is to be submitted using the NHG DSRB Study Status Report Form within 4 to 6 weeks of study completion.

With the enactment of the Human Biomedical Research Act, Health Products Act, Medicines Act and their subsidiary legislations, Principal Investigators are reminded to ensure that their research complies with the regulatory requirements stipulated in the applicable Acts. Contraventions under any of these Acts are criminal offences and would result in fines or imprisonment or both, subject to the nature of the offence.

Established since May 2006, the NHG Research Quality Management (RQM) Programme seeks to promote the responsible conduct of research in a research culture with high ethical standards, identify potential systemic weaknesses and make recommendations for continual improvement. Hence, this

research study may be randomly selected for a review by the Research Quality Management (RQM) team. For more information, please visit www.research.nhg.com.sg.

The NHG DSRB operates in accordance to the ICH GCP and all applicable laws and regulations.

Yours Sincerely

A/Prof Patrick Chan

Chairman

NHG Domain Specific Review Board D

Cc: Institutional Representative, NUH

c/o Research Office, NUH

Departmental Representative of Surgery, NUH

(This is an electronic-generated letter. No signature is required.)

NHG DSRB – DOMAIN D MEMBERSHIP LIST

(Term from April 2022 to March 2024) / Quorum for Domain: 5

STUDY TITLE: A pilot open-label randomised trial comparing the effectiveness of steroids and methotrexate for the treatment of idiopathic granulomatous mastitis

Date of the Online Meeting at which the application as titled above was reviewed: 07 December 2023

MEMBER'S NAME	PRIMARY SCIENTIFIC / NON-SCIENTIFIC SPECIALTY	INSTITUTION	MALE / FEMALE	PRESENT FOR THE MEETING

A/Prof Patrick Chan Mun Yew (Chairman)	General Surgery	Tan Tock Seng Hospital	M	No
A	Obstetrics & Gynaecology	National University Hospital	F	No
B	Urology	National University Hospital	M	No
C	Orthopaedics	Mount Elizabeth Medical Centre	M	No
D	Orthopaedic Spine & Hand Surgery	Mount Elizabeth Medical Centre	M	No
E	Rehabilitation	Ng Teng Fong General Hospital	M	No
F	Physiotherapy	SIT	M	Yes
G	Anaesthesia	Woodlands Health Campus	M	Yes
I	Surgery	National University Hospital	M	No
J	Sociology	Duke-NUS	F	Yes
K	Biostatistics, Epidemiology, Health Policy	National University Singapore	M	No
L	Quality Management	Khoo Teck Puat Hospital	F	Yes
M	Legal Studies	Tan Tock Seng Hospital	F	No
N	Business Policy	National University Singapore	F	Yes
O	Administration and Operations	Singapore Institute of Technology	M	Yes
P	Social Services	AMKFSC Community Services Ltd	F	Yes
Q	Public Health	NHG HQ	F	No

The NHG DSRB operates in accordance to the ICH GCP and all applicable laws and regulations. Please note that it is the NHG DSRB policy to not reveal the names of the members of the Board. Please be assured that members of the Board with any potential conflict of interest for a particular study abstained from decision making and voting for that study.
